# Association of Android and Gynoid Fatness With Incident Dementia and Brain Structure

**DOI:** 10.1002/jcsm.70095

**Published:** 2025-10-14

**Authors:** Zhong‐Yue Liu, Yu‐Wen Qian, Ji‐Mei Gu, Xiao‐Ping Shao, Meng‐Yuan Miao, Jie‐Qiong Lyu, Zhong‐Xiao Wan, Li‐Qiang Qin, Qi Fang, Guo‐Chong Chen

**Affiliations:** ^1^ Department of Neurology, The Fourth Affiliated Hospital (Medical Center of Soochow University), School of Public Health, Suzhou Medical College Soochow University Suzhou China; ^2^ MOE Key Laboratory of Geriatric Diseases and Immunology, Suzhou Medical College Soochow University Suzhou China; ^3^ Department of Clinical Nutrition The First People's Hospital of Kunshan Suzhou China

**Keywords:** adiposity, android fat, brain MRI, dementia, gynoid fat

## Abstract

**Background:**

The relationship between regional adiposity and dementia remains poorly understood.

**Methods:**

This study included 440 861 UK Biobank participants initially free of dementia, stroke and cancer. Hazard ratios (HRs) and 95% confidence intervals (CIs) of dementia across quartiles of waist circumference (WC) or hip circumference (HC) were calculated, after multivariable adjustment with mutual adjustment for WC and HC. The potential mediating roles of 21 biomarkers covering distinct metabolic pathways were assessed. In a subsample with body composition quantified by dual‐energy X‐ray absorptiometry, the associations of android and gynoid fatness with magnetic resonance imaging‐derived volumetric brain measures were further assessed.

**Results:**

Over a median follow‐up of 12.7 years, there were 2899 incident cases of dementia in females and 3306 cases in males. After multivariable adjustment, WC was positively associated with the risk of dementia (predominantly vascular dementia) in both sexes, but the associations disappeared after further adjusting for the treatments for metabolic disorders. HC was inversely associated with dementia both in females (HR_Q4 vs. Q1_ = 0.75; 95% CI: 0.64, 0.86; *P*‐trend < 0.001) and in males (HR_Q4 vs. Q1_ = 0.83; 95% CI: 0.72, 0.95; *P*‐trend = 0.018), with the leading mediator being apolipoprotein B (11.15%) and vitamin D (9.03%) for female and male associations, respectively. In both sexes, gynoid fat percent was related to larger grey matter volumes (β _female_ = 1.21; 95% CI: 0.74, 1.68; β _male_ = 0.96; 95% CI: 0.28, 1.64) and smaller volumes of white matter hyperintensities (β _female_ = −1.96; 95% CI: −2.50, −1.43; β _male_ = −2.57; 95% CI: −3.39, −1.76).

**Conclusions:**

Android and gynoid adipose tissues may exert divergent influences on the development of dementia, with evidence for shared and distinct mechanisms between sexes.

## 1 Introduction

Dementia is a substantial public health problem worldwide. Due to the ongoing population aging, the absolute number of cases of dementia is estimated to mostly triple between 2019 and 2050, to a staggering 152.8 million living with dementia in 2050, with the highest rise in developing countries [1]. The 2024 report of the *Lancet* standing Commission highlighted the crucial role of 14 modifiable risk factors, including obesity, in the early prevention of dementia [2].

Obesity is an established risk factor for diabetes, and many other chronic diseases (e.g., hypertension and ischaemic stroke) that increase the risk of dementia [2, 3]. Nevertheless, previous studies, which mostly used body mass index (BMI) as a marker of general obesity, have put forward inconsistent conclusions regarding the relationship between obesity and dementia [4]. It is now widely acknowledged that BMI does not necessarily reflect the amount and location of adipose tissue [5], especially in older adults who often experience sarcopenic obesity and preferential visceral fat accumulation [6, 7], which are poorly captured by using BMI alone.

The biological functions of adipose tissue are location‐dependent in terms of various metabolic processes, which may affect dementia risk [8, 9]. As compared with android adiposity, the subcutaneous adipose tissue in the gynoid region fares better regarding the impact on common cardiometabolic risk factors [8]. Thus, anthropometric measures of regional adiposity may offer significant value in addressing the limitation of BMI in assessing fat distribution. Waist circumference (WC) is a well‐validated surrogate for central/android fat accumulation, and hip circumference (HC) serves as a practical indicator of gynoid fat deposition [10]. To this point, the independent effects of regional adiposity on dementia development, particularly the potential protective role of gynoid fat, remain inadequately investigated.

Using data from the UK Biobank (UKB), we assessed the associations of regional adiposity with the risk of dementia. We first examined the prospective associations of WC (as a surrogate measure of android fat) and HC (gynoid fat) with the long‐term risk of dementia and dementia subtypes, taking into account the potential mediating role of a comprehensive set of biomarkers covering distinct biological pathways. Moreover, the widespread availability and recent advances in imaging technologies have significantly enhanced the role of neuroimaging measures in elucidating the underlying aetiologies of dementia [11, 12, 13]. Thus, in a subsample with body composition quantified by dual‐energy X‐ray absorptiometry (DXA), we assessed android and gynoid fatness in relation to magnetic resonance imaging (MRI)‐derived volumetric brain measures.

## 2 Methods

### 2.1 Study Design and Population

The UKB is a large population cohort that captures the population from middle to old age and is well suited for studying conditions that arise in aging, such as cognitive impairments and dementia. The UKB performed the baseline recruitment of more than 500 000 participants during 2006–2010. The first imaging assessment during the follow‐up began in 2014 and is ongoing. The UKB received ethical approval from the research ethics committee (reference for UK Biobank 11/NW/0382) and participants provided written informed consent.

**FIGURE 1:**
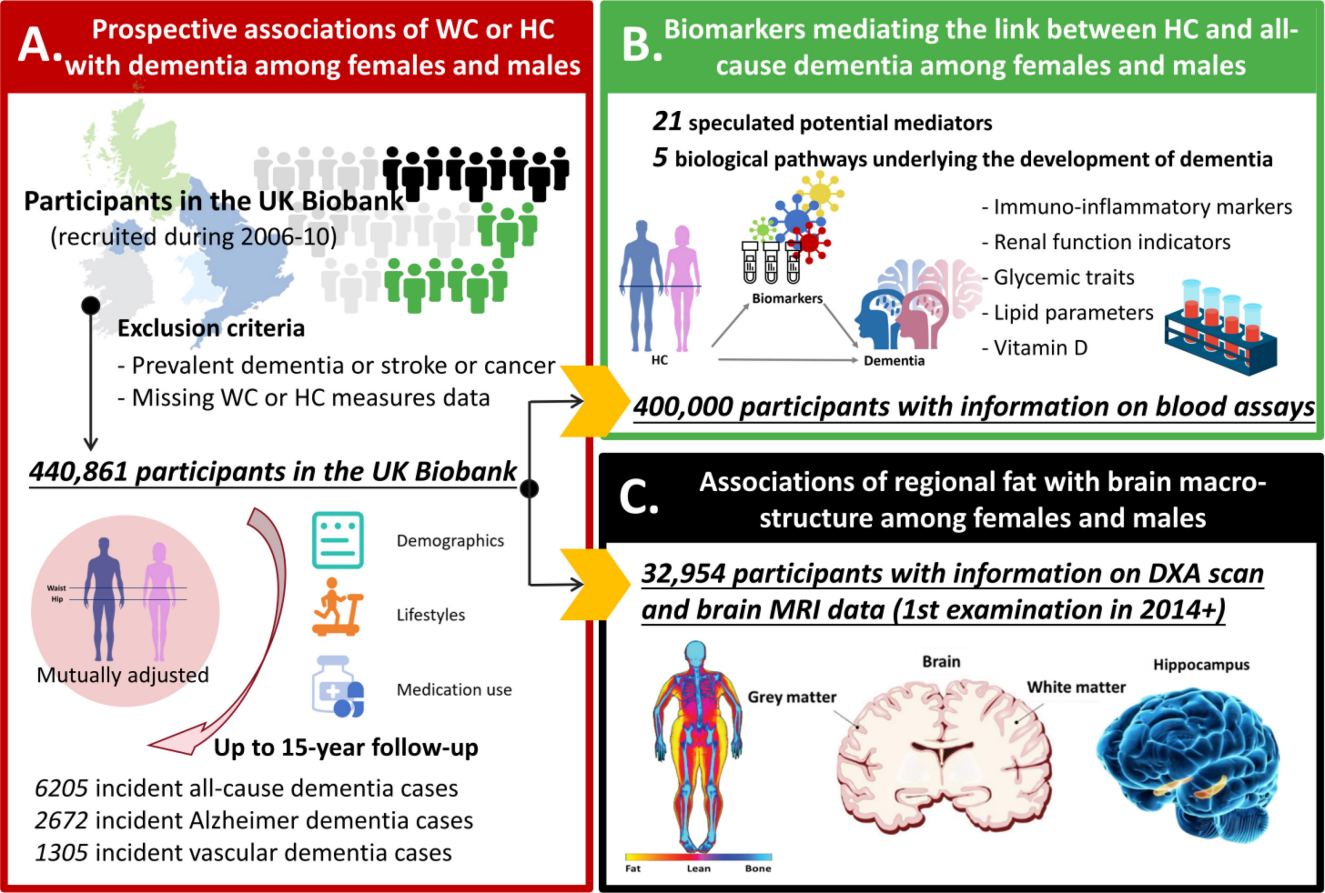
Study design and participant inclusion process. DXA, dual‐energy X‐ray absorptiometry; HC, hip circumference; MRI, magnetic resonance imaging; WC, waist circumference.

For the longitudinal analysis investigating baseline WC or HC and incident dementia, 440 861 participants were included after excluding participants with prevalent dementia, stroke or cancer at baseline, as well as those missing WC or HC measurements. Among these, 400 000 participants with additional measurements of various blood assays at baseline were included in the subsequent mediation analyses exploring biological mechanisms. A subsample (approximately 8.0%) of the primary cohort attended the first imaging examination. For the cross‐sectional analyses on DXA‐derived regional body fat and neuroimaging measures, we further excluded individuals who had developed dementia, stroke or cancer by the time of this imaging visit, including 32 954 participants. Accordingly, covariates for these cross‐sectional analyses were updated using data collected during the first imaging examination (Figure 1).

### 2.2 Anthropometric and Body–Composition Measurements

Anthropometric variables such as height, WC and HC were measured by trained staff using standard procedures, and the details have been described elsewhere [16]. DXA (GE‐Lunar, Madison, WI) scans were performed by a trained radiographer to obtain body–composition measures (https://biobank.ndph.ox.ac.uk/showcase/refer.cgi?id=502) [17]. Numerical measurements of fat mass were directly transferred from the DXA instrument to UKB servers, with no postprocessing. In the present analysis, relative body‐fat measures, defined as the percentage of regional fat mass (i.e., android and gynoid) relative to the total fat mass of the whole body, were evaluated. Conventional anthropometric measures have been found to be strongly correlated with imaging‐derived measures of body fat [10]. In the first UKB imaging study with both types of measures available, WC correlated with android fat (r _Pearson_ = 0.88 in females and 0.89 in males), and HC correlated with gynoid fat (r _Pearson_ = 0.89 in females and 0.82 in males) identically.

### 2.3 Assessments of Covariates and Biomarkers

At baseline, information on sociodemographic factors, lifestyles (e.g., smoking, alcohol drinking, physical activity and sedentary behaviours) and medication usage was collected using touchscreen‐based questionnaires and nurse‐led interviews. The Townsend deprivation index, a composite measure of social deprivation, was derived based on four socioeconomic variables (unemployment, noncar ownership, nonhome ownership and household overcrowding).

According to the prior knowledge on the biological pathways underlying the development of dementia, 21 biomarkers within five biological pathways were considered (Table S1). These included seven immuno‐inflammatory markers, two renal function indicators, three glycaemic traits, seven lipid parameters and vitamin D. Most of these biomarkers were directly quantified, whereas several indices were computed through specific calculations (Supporting Information). The estimated glomerular filtration rate was calculated based on cystatin C values [14]. Composite indices like the low‐grade inflammation score (INFLA‐score) [15] and the systemic immune‐inflammation index (SII) [16] may offer a more comprehensive evaluation of chronic inflammatory status or peripheral immunity with multiple markers incorporated, addressing the limitation of a single marker.

### 2.4 Ascertainments of Incident Dementia and Volumetric Brain Measures

The primary outcome was the first occurrence of any dementia events after baseline recruitment. The secondary outcomes included two major subtypes for dementia, namely, Alzheimer dementia (ad) and vascular dementia (VaD). All these outcomes were ascertained using the algorithmically defined outcome resource provided by UKB (data‐field 42018‐42023). This algorithm systematically integrates the coded information from UKB's baseline assessment data collection (i.e., self‐reported) along with linked data from hospital admissions (HES APC/SMR01/PEDW) and death registries (primary/contributory causes). The diagnosis date of dementia was determined by the earliest evidence of a relevant code in any of these sources. A full list of the ICD and self‐report codes used can be found in https://biobank.ndph.ox.ac.uk/showcase/showcase/docs/alg_outcome_dementia.pdf. It has been proven that the algorithm successfully identifies the large majority of true dementia cases within the cohort, with the positive predictive value typically exceeding 80% [17, 18].

UKB acquired brain T1‐ and diffusion‐weighted MRI data using a 3T Siemens Skyra scanner. The brain MRI information was then processed by the UKB team and made available to approved researchers as imaging‐derived phenotypes (IDPs) [19]. The current analysis included 6 IDPs. Total brain volume (TBV) was computed as the sum of grey and white matter volumes (WMV). The other 5 IDPs, including WMV, grey matter volume (GMV), hippocampus volume (HV), hippocampal grey matter volume (HGMV) and white matter hyperintensities volume (WMHV), were normalized for head size by multiplying the raw IDP by the T1‐based ‘head size scaling factor’ [19].

### 2.5 Statistical Analysis

Considering the potential sex difference in the adiposity–brain–cognition axis [20, 21], all analyses were performed for females and males separately. Baseline participant characteristics were presented according to incident dementia status. Data were presented as mean (standard deviation [SD]) for continuous variables and as number (percentage) for categorical variables.

Cox proportional hazards models were used to estimate hazard ratios (HRs) and 95% confidence intervals (CIs) of incident dementia according to quartiles of WC or HC. Results from the Schoenfeld residuals method suggested that there was no evidence of violation of the proportional hazard assumption. Follow‐up time was counted as the time interval between inception date and the date of diagnosis of dementia, loss to follow‐up, death or the most recent follow‐up for the approved dataset (12 November 2021), whichever came first. Three Cox models were constructed to account for potential confounders. The first model included age at baseline, race/ethnicity, education, Townsend deprivation index, height and WC (for the analyses on HC) or HC (for the analyses on WC). The second model further included smoking status, drinking status, sedentary time and physical activity. The final model included all covariates in Model 2 and further included use of antihypertensive medication, lipid‐lowering medication, diabetes medication and nonsteroidal anti‐inflammatory drugs (NSAIDs). We adjusted for height but not body weight to account for total body size while mitigating multicollinearity and potential overadjustment bias [22, 23].

Potential nonlinear relationships between WC or HC and dementia risk were examined using 4‐knot restricted cubic splines. A *p*‐value for nonlinearity was calculated by testing the null hypothesis that the coefficient of the second spline was equal to 0. Several sensitivity analyses were performed to ensure the robustness of our findings. First, we excluded dementia cases diagnosed within 2 years since enrollment to avoid potential reverse causation. Second, we excluded participants with sex‐specific extreme values (top or bottom 5%) of WC or HC to minimize the potential impact of outliers. Third, to address the correlations between WC and HC (*r* = 0.73), we derived predicted WC or HC and assessed their associations with dementia risk. We regressed measured WC against HC, age and race/ethnicity using linear regression and then summed the yielded participant‐specific residuals with sex‐specific mean WC to calculate the predicted WC. The predicted HC was derived using a similar approach. Fourth, as both body fat distribution and incidence of dementia are age‐dependent, we conducted a stratified analysis by age group (< 50 years vs. ≥ 50 years at baseline).

Pearson partial correlation coefficients were calculated between WC and HC and the aforementioned 21 biomarkers, with adjustment for age, race/ethnicity and WC (for the analyses on HC) or HC (for WC). Mediation analyses were performed to investigate whether these biomarkers potentially mediate the examined relationships. Bonferroni corrections were applied to assess significant indirect effects, taking into account the groups (pathways) of biomarkers tested (*n* = 5).

In addition, we used multivariable linear regression models to assess the cross‐sectional associations of android and gynoid fat percentages with brain structural measurements. All 6 IDPs were standardized and the WMHV was log‐transformed to normalize their distributions. The covariates were the same as in the primary analysis and were measured at the time of the first imaging examination.

All statistical tests were two‐sided and analyses were performed using Stata (version 17.1; StataCorp), SAS (release 9.4; SAS Institute Inc.) and R (version 4.3.2; R Foundation).

## 3 Results

### 3.1 Participant Characteristics

A total of 440 861 participants, aged 37 to 73 years, were eligible for the primary analysis. The mean (SD) age was 56.1 (8.1) years and 234 569 (53.2%) were female. Sex‐specific participant characteristics by incident dementia status are presented in Table S2. For both sexes, as compared with the dementia‐free participants, those who developed dementia tended to be older, had higher levels of social deprivation and lower levels of compulsory educational attainment, were less likely to be never smokers and were more likely to receive treatments for metabolic disorders (i.e., hypertension, dyslipidaemia or diabetes). Moreover, participants with incident dementia had higher levels of both measured and predicted WC, had a comparable measured HC, but had a smaller predicted HC (Table S2).

### 3.2 Associations of WC and HC With Risk of Dementia

Median follow‐up was 12.7 years for both sexes. There were 2899 incident cases of dementia (including 1357 ad and 531 VaD cases) among females and 3306 cases of dementia (1315 ad and 774 VaD cases) among males.

With adjustment for sociodemographic factors, height and HC (Model 1), for both sexes, WC was positively associated with the risk of all‐cause dementia and especially VaD, but not ad (Table 1). For both sexes, the associations between WC and all‐cause dementia or VaD were attenuated after the additional adjustment for lifestyle factors (Model 2). Further adjustment for medication use (Model 3) completely diminished these associations, with HRs of all‐cause dementia of 1.03 (95% CI: 0.88, 1.21) in females (*P*‐trend = 0.89) and 0.96 (95% CI: 0.83, 1.11) in males (*P*‐trend = 0.26) comparing the highest with the lowest sex‐specific quartiles of WC. In males, there was an unexpected inverse association between WC and the risk of ad after the full adjustment (HR _Q4 vs. Q1_ = 0.76; 95% CI: 0.60, 0.96; *P*‐trend = 0.017).

**TABLE 1:** Prospective associations of waist circumference with dementia and dementia subtypes among females and males.

	Quartiles of waist circumference				*P*‐trend	Each SD increment
	Q1	Q2	Q3	Q4		
**Female**						
Range (median), cm	< 75 (71)	75 to < 84 (80)	84 to < 92 (88)	≥ 92 (100)		
**Dementia**						
Model 1	Ref.	1.05 (0.94–1.19)	1.08 (0.94–1.23)	1.34 (1.15–1.56)	< 0.001	1.21 (1.14–1.29)
Model 2	Ref.	1.04 (0.93–1.17)	1.04 (0.91–1.19)	1.25 (1.07–1.46)	0.007	1.17 (1.09–1.25)
Model 3	Ref.	1.00 (0.89–1.12)	0.95 (0.83–1.09)	1.03 (0.88–1.21)	0.89	1.06 (0.99–1.13)
**Alzheimer dementia**						
Model 1	Ref.	1.00 (0.85–1.19)	0.65 (0.79–1.15)	1.15 (0.92–1.44)	0.26	1.17 (1.06–1.29)
Model 2	Ref.	1.00 (0.85–1.18)	0.94 (0.77–1.13)	1.10 (0.88–1.38)	0.46	1.14 (1.04–1.26)
Model 3	Ref.	0.96 (0.81–1.13)	0.86 (0.71–1.04)	0.92 (0.73–1.16)	0.39	1.04 (0.95–1.15)
**Vascular dementia**						
Model 1	Ref.	1.14 (0.84–1.55)	1.38 (1.00–1.92)	1.94 (1.34–2.81)	< 0.001	1.33 (1.14–1.54)
Model 2	Ref.	1.12 (0.82–1.51)	1.32 (0.95–1.84)	1.76 (1.21–2.55)	0.003	1.26 (1.09–1.46)
Model 3	Ref.	1.03 (0.76–1.40)	1.13 (0.81–1.57)	1.28 (0.87–1.87)	0.26	1.08 (0.93–1.26)
**Male**						
Range (median), cm	< 90 (85)	90 to < 97 (93)	97 to < 104 (100)	≥ 104 (110)		
**Dementia**						
Model 1	Ref.	1.02 (0.91–1.13)	1.04 (0.92–1.18)	1.22 (1.05–1.40)	0.014	1.15 (1.08–1.22)
Model 2	Ref.	1.00 (0.90–1.11)	1.00 (0.89–1.14)	1.12 (0.97–1.30)	0.18	1.11 (1.04–1.18)
Model 3	Ref.	0.95 (0.86–1.06)	0.92 (0.81–1.04)	0.96 (0.83–1.11)	0.40	1.02 (0.96–1.09)
**Alzheimer dementia**						
Model 1	Ref.	0.94 (0.80–1.11)	0.96 (0.79–1.16)	0.90 (0.71–1.13)	0.33	0.97 (0.88–1.07)
Model 2	Ref.	0.94 (0.80–1.11)	0.95 (0.79–1.15)	0.88 (0.70–1.10)	0.23	0.96 (0.87–1.06)
Model 3	Ref.	0.90 (0.76–1.06)	0.88 (0.74–1.07)	0.76 (0.60–0.96)	0.017	0.89 (0.80–0.99)
**Vascular dementia**						
Model 1	Ref.	1.15 (0.90–1.45)	1.31 (1.00–1.71)	1.92 (1.42–2.57)	< 0.001	1.47 (1.30–1.66)
Model 2	Ref.	1.10 (0.87–1.39)	1.21 (0.93–1.58)	1.66 (1.23–2.25)	0.001	1.37 (1.21–1.55)
Model 3	Ref.	1.00 (0.79–1.27)	1.02 (0.78–1.33)	1.21 (0.89–1.64)	0.26	1.16 (1.02–1.32)

*Note:* Data are presented as hazard ratio (HR) and 95% confidence interval (CI). Waist circumference and hip circumference were mutually adjusted for each other (in quartile). **Model 1** was adjusted for age at baseline (continuous, years), race/ethnicity (White, non‐White and unknown), educational level (< 9, 9 to < 12, 12 to < 16, ≥ 16 years and unknown), Townsend deprivation index (continuous) and height (continuous, cm). **Model 2** was further adjusted for smoking status (never, former, current: < 10, 10 to < 50, ≥ 50 pack‐years and unknown), drinking status (never, former, current: 0, < 0.5, 0.5 to < 1, ≥ 1 drinks/day and unknown), sedentary time (continuous, h/day) and physical activity (continuous, MET‐h/week). **Model 3** was further adjusted for use of antihypertensive medication (yes, no), lipid‐lowering medication (yes, no), diabetes medication (yes, no) and NSAIDs (yes, no).

Abbreviation: SD, standard deviation.

In contrast, after the full adjustment, HC remained significantly and inversely associated with the risk of all‐cause dementia both in females (HR _Q4 vs. Q1_ = 0.75; 95% CI: 0.64, 0.86; *P*‐trend = 0.001) and in males (HR _Q4 vs. Q1_ = 0.83; 95% CI: 0.72, 0.95; *P*‐trend = 0.018). In females, the association of HC with the risk of ad was stronger than the association for VaD, whereas in males, the association of HC with the risk of VaD appeared to be more evident than that for ad (Table 2).

**TABLE 2:** Prospective associations of hip circumference with dementia and dementia subtypes among females and males.

	Quartiles of hip circumference				*P*‐trend	Each SD increment
	Q1	Q2	Q3	Q4		
**Female**						
Range (median), cm	< 96 (93)	96 to < 102 (100)	102 to < 108 (105)	≥ 108 (115)		
**Dementia**						
Model 1	Ref.	0.76 (0.68–0.85)	0.74 (0.65–0.84)	0.72 (0.62–0.83)	< 0.001	0.85 (0.80–0.91)
Model 2	Ref.	0.77 (0.69–0.86)	0.75 (0.66–0.85)	0.71 (0.61–0.82)	< 0.001	0.85 (0.80–0.91)
Model 3	Ref.	0.78 (0.70–0.87)	0.78 (0.69–0.89)	0.75 (0.64–0.86)	0.001	0.89 (0.83–0.94)
**Alzheimer dementia**						
Model 1	Ref.	0.73 (0.62–0.85)	0.73 (0.60–0.85)	0.63 (0.51–0.78)	< 0.001	0.78 (0.70–0.86)
Model 2	Ref.	0.73 (0.62–0.85)	0.73 (0.61–0.88)	0.62 (0.50–0.77)	< 0.001	0.78 (0.70–0.86)
Model 3	Ref.	0.74 (0.63–0.87)	0.76 (0.63–0.91)	0.65 (0.52–0.81)	< 0.001	0.81 (0.73–0.89)
**Vascular dementia**						
Model 1	Ref.	0.81 (0.61–1.06)	0.64 (0.47–0.89)	0.80 (0.57–1.12)	0.43	0.94 (0.81–1.08)
Model 2	Ref.	0.82 (0.63–1.08)	0.67 (0.48–0.92)	0.82 (0.58–1.16)	0.52	0.96 (0.83–1.11)
Model 3	Ref.	0.85 (0.65–1.11)	0.71 (0.52–0.98)	0.88 (0.62–1.24)	0.75	1.01 (0.87–1.17)
**Male**						
Range (median), cm	< 99 (96)	99 to < 104 (101)	104 to < 108 (105)	≥ 108 (111)		
**Dementia**						
Model 1	Ref.	0.83 (0.75–0.92)	0.77 (0.68–0.87)	0.80 (0.69–0.92)	0.006	0.89 (0.83–0.94)
Model 2	Ref.	0.85 (0.77–0.94)	0.79 (0.70–0.90)	0.82 (0.71–0.94)	0.014	0.90 (0.85–0.96)
Model 3	Ref.	0.86 (0.78–0.95)	0.81 (0.71–0.92)	0.83 (0.72–0.95)	0.018	0.92 (0.86–0.98)
**Alzheimer dementia**						
Model 1	Ref.	0.86 (0.73–1.01)	0.78 (0.64–0.96)	0.89 (0.71–1.11)	0.34	0.97 (0.88–1.07)
Model 2	Ref.	0.87 (0.74–1.01)	0.79 (0.65–0.97)	0.90 (0.72–1.12)	0.36	0.97 (0.88–1.07)
Model 3	Ref.	0.88 (0.75–1.03)	0.81 (0.66–0.99)	0.90 (0.72–1.13)	0.40	0.99 (0.90–1.09)
**Vascular dementia**						
Model 1	Ref.	0.70 (0.56–0.87)	0.67 (0.51–0.87)	0.67 (0.50–0.89)	0.032	0.78 (0.69–0.89)
Model 2	Ref.	0.72 (0.58–0.90)	0.70 (0.54–0.92)	0.70 (0.52–0.93)	0.06	0.81 (0.71–0.91)
Model 3	Ref.	0.74 (0.60–0.93)	0.74 (0.59–0.96)	0.71 (0.53–0.94)	0.06	0.84 (0.74–0.95)

*Note:* Data are presented as hazard ratio (HR) and 95% confidence interval (CI). Waist circumference and hip circumference were mutually adjusted for each other (in quartile). **Model 1** was adjusted for age at baseline (continuous, years), race/ethnicity (White, non‐White and unknown), educational level (< 9, 9 to < 12, 12 to < 16, ≥ 16 years and unknown), Townsend deprivation index (continuous) and height (continuous, cm). **Model 2** was further adjusted for smoking status (never, former, current: < 10, 10 to < 50, ≥ 50 pack‐years and unknown), drinking status (never, former, current: 0, < 0.5, 0.5 to < 1, ≥ 1 drinks/day and unknown), sedentary time (continuous, h/day) and physical activity (continuous, MET‐h/week). **Model 3** was further adjusted for use of antihypertensive medication (yes, no), lipid‐lowering medication (yes, no), diabetes medication (yes, no) and NSAIDs (yes, no).

Abbreviation: SD, standard deviation.

**FIGURE 2:**
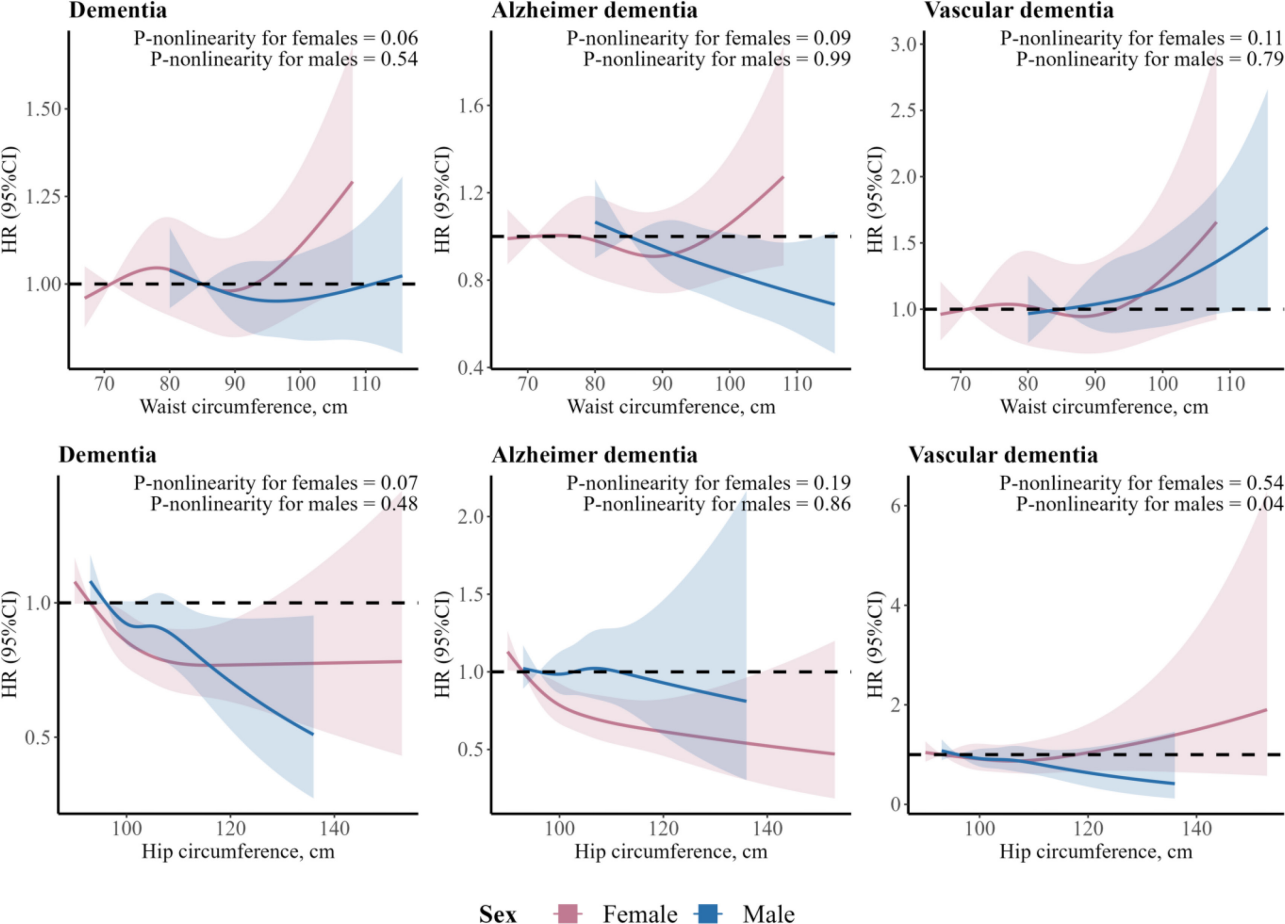
Restricted cubic splines for the associations of waist or hip circumference with all‐cause dementia and dementia subtypes among females and males. Results were adjusted for covariates listed for Model 3 in Table 1. Waist circumference and hip circumference were mutually adjusted for each other as continuous variables. The splines were modelled with four knots, and the median of the first quartile level of waist or hip circumference (stratified by sex) was used as the reference.

There was little evidence for nonlinear associations of WC or HC with risk of dementia or its subtypes (all *P*‐nonlinearity > 0.05) (Figure 2). The major findings from the main analyses remained robust in a number of predefined sensitivity analyses (Tables S3–S5). In age‐stratified analysis, the direction and magnitude of the associations observed in participants aged ≥ 50 years largely aligned with the findings from our primary analysis of the entire cohort, whereas the associations in the younger group were generally nonsignificant, likely due to the low statistical power resulting from the limited number of dementia cases (57 cases in females and 66 cases in males) (Tables S6–S7).

### 3.3 Role of Biomarkers in Mediating the Link Between HC and Dementia

**FIGURE 3:**
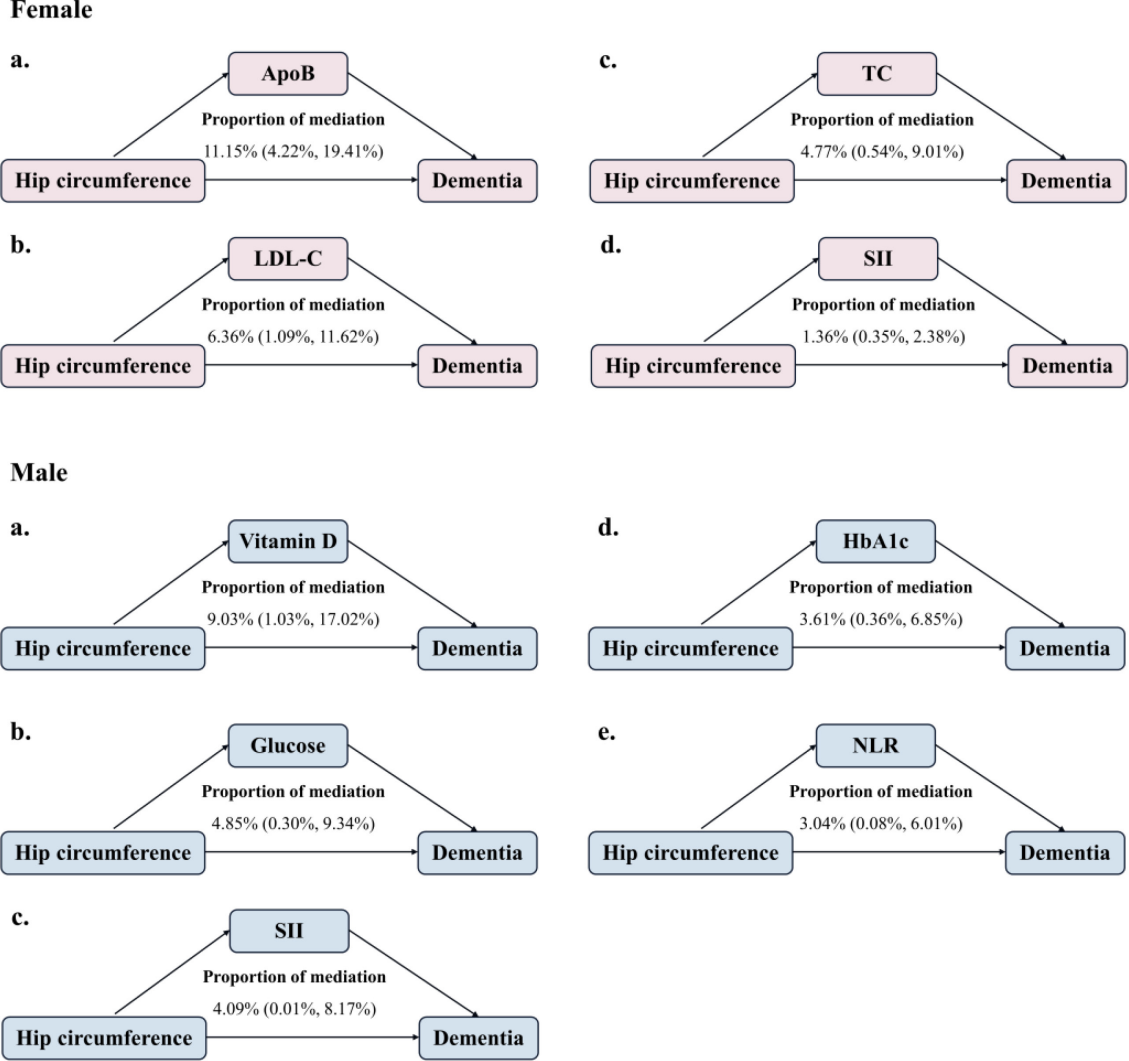
Mediation of biomarkers in the associations of hip circumference and all‐cause dementia among females and males. Results were adjusted for covariates listed for Model 3 in Table 1. Hip circumference, waist circumference and biomarkers were standardized by *z*‐score. ApoB, apolipoprotein B; HbA1c, glycosylated haemoglobin; LDL‐C, low‐density lipoprotein cholesterol; NLR, neutrophil to lymphocyte ratio; SII, systemic immune‐inflammation index; TC, total cholesterol.

Correlations between WC or HC and the speculated potential mediators among females and males are presented in Figure S1. In general, WC (|*r*| < 0.40) showed stronger correlations with these biomarkers than did HC (|*r*| < 0.25). However, the correlations between HC and these biomarkers appeared to be more sex‐specific than the correlations for WC. We proceeded to evaluate the potential mediating effects of each of the 21 biomarkers on the associations between HC and dementia, after adjusting for covariates in Model 3. For the association in females, the Top 3 mediators were apolipoprotein B (ApoB) (11.15%), low‐density lipoprotein cholesterol (LDL‐C) (6.36%) and total cholesterol (TC) (4.77%). For the male association, vitamin D (9.03%) and glucose (4.85%), followed by SII (4.09%), were the leading mediators (Figure 3).

### 3.4 Association of Android and Gynoid Fatness With Brain Structure

**FIGURE 4:**
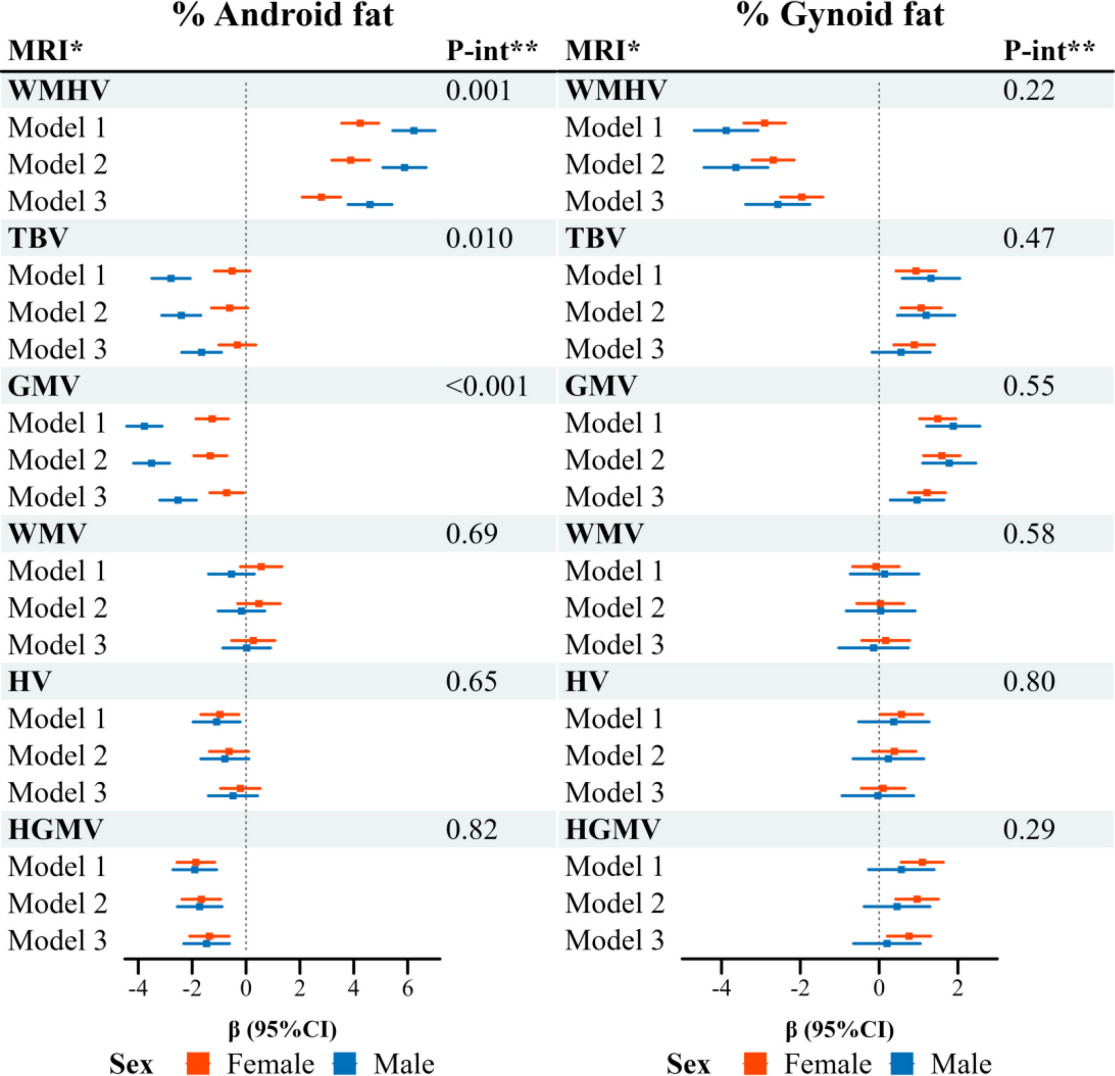
Forest plot for the associations of android or gynoid body fat percent with brain structural measurements among females and males.*Units of brain measurements are mm^3^. **P‐int represents the interaction *p*‐value for sex with android or gynoid fat percent in Model 3. WMHV was natural log‐transformed. All brain structural measurements were standardized by *z*‐score. GMV, grey matter volume; HGMV, hippocampal grey matter volume; HV, hippocampus volume; TBV, total brain volume; WMHV, white matter hyperintensity volume; WMV, white matter volume; **Model 1** was adjusted for age at baseline (continuous, years), race/ethnicity (White, non‐White and unknown), educational level (< 9, 9 to< 12, 12 to < 16, ≥ 16 years and unknown), Townsend deprivation index (continuous) and height (continuous, cm). **Model 2** was further adjusted for smoking status (never, former, current: < 10, 10 to < 50, ≥ 50 pack‐years and unknown), drinking status (never, former, current: 0, < 0.5, 0.5 to < 1, ≥ 1 drinks/day and unknown) and physical activity (continuous, MET‐h/week). **Model 3** was further adjusted for use of antihypertensive medication (yes, no), lipid‐lowering medication (yes, no), diabetes medication (yes, no) and NSAIDs (yes, no).

After the full adjustment, higher android fat percent was significantly associated with a larger WMHV and a smaller GMV and HGMV in both sexes, with the associations for WMHV and GMV being stronger in males than in females (*P*‐interaction < 0.01). Higher android fat percent was associated with a larger TBV in males only (*P*‐interaction < 0.001) (Figure 4 and Table S8). Higher gynoid fat percent was significantly associated with a smaller WMHV and a larger GMV, similarly in females and males (Figure 4). Higher gynoid fat percent was significantly associated with a larger TBV and HGMV in females but not in males, whereas the sex differences were not significant (*P*‐interaction > 0.20) (Figure 4 and Table S8).

## 4 Discussion

In the present analysis of UKB participants, we used waist and hip sizes as proxy markers of android and gynoid fatness, respectively, and assessed their relationships with the long‐term risk of dementia. A larger waist size was associated with a higher risk of dementia, whereas a larger hip size was associated with a lower risk of dementia. The waist–dementia association appeared to be interpretable by known risk factors, especially the treatments for common metabolic disorders. However, the inverse hip–dementia association was independent of conventional risk factors. Further mediation analyses suggested that the leading biomarkers partially mediating the link between gynoid fat and dementia varied between sexes, potentially suggesting sex‐specific mechanisms. In a subset of participants with DXA‐derived body fat mass, relatively higher android fat was associated with greater brain atrophy (both in GMV and HGMV) and a larger WMHV, whereas higher gynoid fat was related to a larger GMV and a smaller WMHV.

Midlife obesity defined by BMI has been prudently identified as a risk factor for dementia based on relatively consistent findings [2]. Only a few studies of different fat compartments and dementia risk have been conducted [24]. Most of the prior studies have highlighted the detrimental role of central obesity [24], while relatively limited evidence is available on the potential effect of subcutaneous adipose tissue in the gynoid region on brain health. Our findings affirmed the deleterious effects of abdominal obesity on the risk of dementia (predominantly VaD) and suggested that such effects may be largely mediated by common metabolic dysfunctions.

As compared with midlife adiposity, adiposity during the late life (defined as older than 50 years) has been less consistently associated with dementia, with some studies showing an unexpected inverse association [25, 26, 27], the so‐called ‘obesity paradox’ that has been observed for other health outcomes such as cardiovascular diseases [28, 29] and premature mortality [30]. While such an obesity paradox for dementia may be attributable to factors such as reverse causality [31, 32] and potential nonlinear effects of body weight on dementia [33, 34], it may also result from neglecting the consideration of different body fat distributions. Gynoid fat, which may have favourable impacts on various metabolic processes [8, 35, 36], usually has strong correlations with android fat and whole‐body fat. A lack of considering gynoid fat may attenuate or even reverse a positive relationship between general or android obesity and health risk. In a longitudinal study of older adults with DXA scans to quantify regional fat mass, whole‐body, gynoid and android fat mass were all inversely associated with the risk of dementia in both sexes [27]; after excluding the first 5 years of follow‐up, only the association for gynoid fat among women remained significant [27]. While these previous findings highlight the impact of reverse causation, the correlation between android and gynoid fat was not taken into account in that study, which may have masked a positive relationship between android fat and dementia. In the present study, measures of regional body fat were mutually adjusted for each other across the analyses, consequently demonstrating the independent effects of regional adiposity.

To our knowledge, this is the first study of DXA‐derived gynoid fat and brain structure in a cohort of the general population. The human brain consists of both white matter and grey matter, each playing crucial roles in brain function and structure [37]. Grey matter atrophy typically indicates loss of neurons or shrinkage of neuronal cell bodies and is often associated with cognitive decline [38]. White matter consists mainly of mainly myelinated axons that connect different brain regions, contributing to information processing or balance and walking [37]. White matter hyperintensities may potentially be potentially caused by vascular processes or neurodegenerative disorders [39], pointing to the pathological processes of VaD and ad, respectively. A notable inverse association was identified between gynoid fat and WMHV, contrasting with the effects of overall obesity or android fat [40]. Additionally, higher gynoid fat was associated with a larger GMV and TBV, but not with WMV, exhibiting a region‐specific alteration, which is possibly due to the higher metabolic activity and greater vulnerability of neuronal cell bodies to metabolic abnormalities such as inflammatory processes [41].

From the perspective of adipose biology, the gluteal depot acts as a metabolic reservoir of excess lipids, which helps prevent ectopic fat deposits in muscles and the liver, as well as excessive visceral fat [35]. It also secretes higher levels of protective cytokines such as adiponectin [36], which may combat oxidative stress [42] and metabolic dysfunctions [43]. Disturbances in lipid metabolism and immune‐inflammatory pathways, where gynoid fat shows favourable impacts, are crucial physiological mechanisms in the development of dementia [16, 44]. In the current study, the Top 3 biomarkers mediating the inverse association between HC and dementia differed between sexes (ApoB, LDL‐C and TC in females; and vitamin D, glucose and SII in males). We therefore posit that lipid metabolism might be involved in the potential pathways from gynoid adiposity to the development of female dementia, while gynoid fat may confer neuroprotection in males partially by mitigating oxidative stress and neuroinflammation. Nevertheless, the identified mediators other than ApoB exhibited weak mediating effects (mediation proportion < 10%), indicating that these biomarkers only explain a small degree of the association and the suggested mechanisms should be considered exploratory and require replicative evidence.

Sexual dimorphisms were evident in both body fat distribution [20] and neuropsychiatric conditions [21, 45], indicating that the risk for diseases associated with obesity (particularly regional obesity) may also have biological drivers that are specific or differentially regulated in one sex compared with the other. In addition to the aforementioned variability in the magnitude of effects of hip size on the risk of dementia and its subtypes across sexes, the correlations between HC and metabolic biomarkers also showed some degree of sexual dimorphism. Norreen‐Thorsen et al. yielded that sex and differential patterns of fat distribution significantly influence the expression and metabolism of ApoB [46], which was consistent with our findings that HC has a stronger inverse correlation with ApoB in females. Our findings highlight the rationale and necessity to account for sex differences in the investigation of the relationships of regional adiposity with brain and cognition.

Besides reinforcing abdominal obesity as a uniform risk factor, our findings suggest the potential protection of gynoid fatness on brain health. It is premature to advocate for selectively increasing gynoid fat as a clinical strategy to reduce dementia risk. Rather, HC serves as a readily measurable biomarker indicative of a potentially protective fat distribution pattern, which alongside other adiposity measures may refine dementia risk stratification, particularly in the elderly. On the other hand, a substantial proportion of the association between gynoid fat and dementia remains unexplained. Thus, future research should focus on uncovering the mechanisms behind this protection and exploring strategies (e.g., lifestyle and pharmacological) to enhance these metabolic pathways for dementia prevention, moving beyond simplistic metrics of overall weight or central fat alone.

This study has several strengths. First, the study utilized data from a large sample with a range of information on demographic, lifestyle and medication use that enabled us to minimize the influence of potential confounding. Additionally, information on a set of metabolic biomarkers allowed us to conduct the exploratory analyses of the biological mechanisms underlying the beneficial impact of gynoid fat. Moreover, utilizing imaging data to examine the association between regional body fat and brain structure added a valuable dimension to our research, helping to establish a more direct correlation between physical and cognitive health parameters. Finally, sexual dimorphism was emphasized in our study design, allowing for a more meticulous understanding and exploration of the association between regional body fat and dementia.

Several limitations should be addressed. First, dementia is a clinical syndrome with a variety of underlying pathologies or subtypes, while the neuroimaging measurements considered in our study may only cover the characteristics of brain atrophy and vascular damage, which were the most typical imaging characteristics of dementia. Further studies with multimodality imaging data utilized (i.e., anatomic structural imaging and physiologic molecular imaging [12]) are warranted to confirm our findings. Second, incident cases of dementia were identified by information on hospital admission and death register data in the UKB, such that some cases with mild symptoms may have been missed [17]. Third, the generalizability of our findings to other ethnic populations remains unclear given that participants in the UKB were largely ethnically White [47, 48].

## 5 Conclusions

Our findings suggest that android and gynoid adipose tissues may exert divergent influences on human brain health and the development of dementia, with evidence for shared and distinct mechanisms between sexes. Our findings may also underscore the importance of accounting for gynoid fat when assessing the cognitive consequences of abdominal adiposity to refine risk stratification.

## Ethics Statement

The UK Biobank study was conducted in accordance with the Helsinki Declaration and received ethical approval from the North West Multi‐centre Research Ethics Committee (REC reference for UK Biobank 11/NW/0382). All participants provided written informed consent at recruitment.

## Conflicts of Interest

The authors declare no conflicts of interest.

## Supporting information


**Table S1:** The selected biomarkers and related biological pathways.
**Table S2:** Baseline characteristics of participants by incident dementia status and sex.
**Table S3:** Associations of waist or hip circumference with all‐cause dementia after excluding cases diagnosed within 2 years since enrollment.
**Table S4:** Associations of waist or hip circumference with all‐cause dementia after excluding participants with sex‐specific top or bottom 5% of waist or hip circumference.
**Table S5:** Associations of predicted waist circumference or predicted hip circumference with all‐cause dementia.
**Table S6:** Associations of waist circumference with all‐cause dementia across different age groups.
**Table S7:** Associations of hip circumference with all‐cause dementia across different age groups.
**Table S8:** Associations of android or gynoid fat percent with brain structural measurements among females and males.
**Figure S1:** Correlations between waist or hip circumference and speculated potential mediators among females and males.

## Data Availability

The UK Biobank data during the current study are available upon application to the UK Biobank (www.ukbiobank.ac.uk/).
