# Supplementary material for: Association of Android and Gynoid Fatness With Incident Dementia and Brain Structure
Source: J Cachexia Sarcopenia Muscle. 2025 Oct 14;16(5):e70095. doi: 10.1002/jcsm.70095 (PMC12521049; doi:10.1002/jcsm.70095)
Supplement: Supplementary file 1 — Table S1: The selected biomarkers and related biological pathways. Table S2: Baseline characteristics of participants by incident dementia status and sex. Table S3: Associations of waist or hip circumference with all‐cause dementia after excluding cases diagnosed within 2 years since enrollment. Table S4: Associations of waist or hip circumference with all‐cause dementia after excluding participants with sex‐specific top or bottom 5% of waist or hip circumference. Table S5: Associations of predicted waist circumference or predicted hip circumference with all‐cause dementia. Table S6: Associations of waist circumference with all‐cause dementia across different age groups. Table S7: Associations of hip circumference with all‐cause dementia across different age groups. Table S8: Associations of android or gynoid fat percent with brain structural measurements among females and males. Figure S1: Correlations between waist or hip circumference and speculated potential mediators among females and males. [file JCSM-16-e70095-s001.docx]

**Supplementary methods**

**Calculations of the composite indicators**

Cystatin C–based estimated glomerular filtration rate (eGFR) was calculated with the Chronic Kidney Disease Epidemiology Collaboration (CKD-EPI) 2012 cystatin C equation. Serum cystatin C concentrations were expressed in mg L^-1^, and sex-specific multipliers were incorporated as recommended by the CKD-EPI consortium (1 for males and 0.932 for females). For each participant, eGFR (mL·min^-1^·1.73 m^2^) was computed as follows: (1) if cystatin C ≤0.8 mg·L^-1^, eGFR = 133 × (cystatin C / 0.8) ^–0.499^ × 0.996 ^Age^ × sex-specific multiplier; (2) if cystatin C >0.8 mg·L^-1^, eGFR = 133 × (cystatin C / 0.8) ^–1.328^ × 0.996 ^Age^ × sex-specific multiplier [1].

The INFLA score quantifies chronic low-grade inflammation by integrating four biomarkers: high-sensitivity C-reactive protein (CRP), white blood cell (WBC) count, platelet count, and the neutrophil-to-lymphocyte ratio (NLR). Extreme outlier (values beyond Q1 – 3×IQR or Q3 + 3×IQR) were excluded to minimize the influence of acute illness or measurement error [2]. For each of the biomarkers, all individuals were ranked and assigned to deciles (1st to 10th) based on their biomarker level. Individuals whose biomarker level fell within the 1st to 4th deciles were assigned a score for that biomarker ranging from –4 (1st decile) to –1 (4th decile). Individuals whose biomarker level fell within the 5th or 6th decile were assigned a score of 0 for that biomarker. Individuals whose biomarker level fell within the 7th to 10th deciles were assigned a score for that biomarker ranging from +1 (7th decile) to +4 (10th decile). The scores assigned for each of the four biomarkers (CRP, WBC, Platelet, NLR) were finally summed to yield the individual’s total INFLA score [3]. The INFLA score is a continuous variable with a potential range from –16 to +16, with higher scores indicating a greater burden of chronic low-grade inflammation across the measured pathways.

The systemic immune inflammation index (SII) was calculated based on the formula: SII = Platelet count × NLR [4].”

**Reference**

1. Inker LA, Schmid CH, Tighiouart H, Eckfeldt JH, Feldman HI, Greene T, Kusek JW, Manzi J, Van Lente F, Zhang YL *et al*: **Estimating glomerular filtration rate from serum creatinine and cystatin C**. *N Engl J Med* 2012, **367**(1):20-29.

2. Fang F, Gu JM, Qian YW, Shao XP, Liu ZY, Ge YY, Chen GC: **Quantity and quality of dietary carbohydrates, low-grade inflammation, and risk of chronic obstructive pulmonary disease and lung function**. *Clin Nutr* 2025, **45**:53-60.

3. Pounis G, Bonaccio M, Di Castelnuovo A, Costanzo S, de Curtis A, Persichillo M, Sieri S, Donati MB, Cerletti C, de Gaetano G, Iacoviello L: **Polyphenol intake is associated with low-grade inflammation, using a novel data analysis from the Moli-sani study**. *Thromb Haemost* 2016, **115**(2):344-352.

4. Zhang YR, Wang JJ, Chen SF, Wang HF, Li YZ, Ou YN, Huang SY, Chen SD, Cheng W, Feng JF *et al*: **Peripheral immunity is associated with the risk of incident dementia**. *Mol Psychiatr* 2022, **27**(4):1956-1962.

**Table S1.** The selected biomarkers and related biological pathways.

| **Biological pathways** | **Biomarkers** |
| --- | --- |
| immuno-inflammatory markers | INFLA-score*, WBC, Plt, NLR, CRP, PLR, SII*, LMR |
| lipid parameters | TG, TC, LDL-C, HDL-C, ApoA, ApoB, Lp(a) |
| glycemic traits | glucose, HbA1c, IGF 1 |
| renal function indicators | eGFR*, UCR |
| vitamin | vitamin D |

*Biomarkers were derived through specific calculations.

ApoA, apolipoprotein A; ApoB, apolipoprotein B; CRP, high-sensitivity C-reactive protein; eGFR, estimated glomerular filtration rate; HbA1c, glycosylated hemoglobin; HDL-C, high-density lipoprotein cholesterol; IGF 1, insulin-like growth factor 1; LDL-C, low-density lipoprotein cholesterol; LMR, lymphocyte to monocyte ratio; Lp(a), lipoprotein(a); NLR, neutrophil to lymphocyte ratio; PLR, platelet to lymphocyte ratio; Plt, platelet count; SII, systemic immune-inflammation index; TC, total cholesterol; TG, triglycerides; UCR, urinary creatinine; WBC, white blood cell count

**Table S2.** Baseline characteristics of participants by incident dementia status and sex.

|  | **Female** | | **Male** | |
| --- | --- | --- | --- | --- |
|  | Non dementia | Dementia | Non dementia | Dementia |
| **No. of participants** | 231,670 | 2899 | 202,986 | 3306 |
| **Demographics** | | | | |
| **Age, years** | 55.89 ± 8.00 | 64.11 ± 4.83 | 56.14 ± 8.19 | 64.06 ± 4.89 |
| **Race/ethnicity** |  |  |  |  |
| White | 217,157 (93.7) | 2766 (95.4) | 190,082 (93.6) | 3143 (95.1) |
| Non-White | 13,394 (5.8) | 112 (3.9) | 11,573 (5.7) | 138 (4.2) |
| Unknown | 1119 (0.5) | 21 (0.7) | 1331 (0.7) | 25 (0.8) |
| **Educational level, years** |  |  |  |  |
| <9 | 73,154 (31.6) | 521 (18.0) | 69,031 (34.0) | 743 (22.5) |
| 9 to <12 | 27,497 (11.9) | 240 (8.3) | 20,960 (10.3) | 272 (8.2) |
| 12 to <16 | 53,364 (23.0) | 605 (20.9) | 37,619 (18.5) | 495 (15.0) |
| ≥16 | 73,154 (31.6) | 1434 (49.5) | 71,243 (35.1) | 1654 (50.0) |
| Unknown | 4501 (1.9) | 99 (3.4) | 4133 (2.0) | 142 (4.3) |
| **Townsend deprivation index** | -1.35 ± 3.03 | -0.96 ± 3.31 | -1.25 ± 3.15 | -0.94 ± 3.31 |
| **Lifestyles** | | | | |
| **Smoking status** |  |  |  |  |
| Never | 139,841 (60.4) | 1568 (54.1) | 100,514 (49.5) | 1309 (39.6) |
| Former | 70,674 (30.5) | 1031 (35.6) | 75,692 (37.3) | 1578 (47.7) |
| Current: <10 pack-years | 3002 (1.3) | 22 (0.8) | 2948 (1.5) | 24 (0.7) |
| Current: 10 to<50 pack-years | 15,942 (6.9) | 207 (7.1) | 20,107 (9.9) | 298 (9.0) |
| Current: ≥50 pack-years | 962 (0.4) | 35 (1.2) | 2492 (1.2) | 66 (2.0) |
| Unknown | 1249 (0.5) | 36 (1.2) | 1233 (0.6) | 31 (0.9) |
| **Drinking status** |  |  |  |  |
| Never | 13,522 (5.8) | 309 (10.7) | 5660 (2.8) | 126 (3.8) |
| Former | 8039 (3.5) | 190 (6.6) | 6790 (3.3) | 227 (6.9) |
| Current: <1 drink/week | 64,745 (27.9) | 886 (30.6) | 32,803 (16.2) | 593 (17.9) |
| Current: 1-2 drinks/week | 59,848 (25.8) | 631 (21.8) | 52,711 (26.0) | 744 (22.5) |
| Current: ≥3 drinks/week | 84,805 (36.6) | 863 (29.8) | 104,287 (51.4) | 1598 (48.3) |
| Unknown | 711 (0.3) | 20 (0.7) | 735 (0.4) | 18 (0.5) |
| **Physical activity, MET-h/week** | 38.87 ± 36.54 | 39.21 ± 37.93 | 44.31 ± 45.59 | 43.08 ± 46.32 |
| **Sedentary time, h/day** | 4.35 ± 2.17 | 4.55 ± 2.39 | 5.30 ± 2.69 | 5.44 ± 2.69 |
| **Medication usage** | | | | |
| **Antihypertensive medication** | 38,347 (16.6) | 977 (33.7) | 46,049 (22.7) | 1375 (41.6) |
| **Lipid-lowering medication** | 27,654 (11.9) | 891 (30.7) | 44,513 (21.9) | 1420 (43.0) |
| **Diabetes medication** | 5234 (2.3) | 211 (7.3) | 8856 (4.4) | 371 (11.2) |
| **NSAIDs** | 40,141 (17.3) | 448 (15.5) | 26,574 (13.1) | 421 (12.7) |
| **Anthropometric measurements** | | | | |
| **Height, cm** | 162.47 ± 6.31 | 160.39 ± 6.30 | 175.71 ± 6.82 | 173.49 ± 6.77 |
| **Waist circumference, cm** | 84.57 ± 12.50 | 87.01 ± 13.15 | 96.82 ± 11.30 | 98.33 ± 12.05 |
| **Hip circumference, cm** | 103.32 ± 10.37 | 103.74 ± 10.70 | 103.42 ± 7.60 | 103.31 ± 8.18 |
| **Predicted waist circumference, cm** | 84.57 ± 7.06 | 86.58 ± 7.48 | 96.82 ± 6.49 | 98.46 ± 6.82 |
| **Predicted hip circumference, cm** | 103.34 ± 5.87 | 102.21 ± 6.10 | 103.44 ± 4.37 | 102.36 ± 4.64 |

Data are presented as mean ± SD for continuous variables and number (%) for categorical variables.

MET, metabolic equivalent; NSAIDs, nonsteroidal anti-inflammatory drugs; SD, standard deviation

**Table S3.** Associations of waist or hip circumference with all-cause dementia after excluding cases diagnosed within 2 years since enrollment.

|  | **Waist circumference** | | | | ***P*-trend** | **Hip circumference** | | | | ***P*-trend** |
| --- | --- | --- | --- | --- | --- | --- | --- | --- | --- | --- |
|  | **Q1** | **Q2** | **Q3** | **Q4** |  | **Q1** | **Q2** | **Q3** | **Q4** |  |
| **Female** | | | | | | | | | | |
| Model 1 | Ref. | 1.03 (0.92-1.16) | 1.05 (0.92-1.20) | 1.29 (1.11-1.51) | <0.001 | Ref. | 0.78 (0.70-0.88) | 0.78 (0.70-0.88) | 0.76 (0.65-0.88) | <0.001 |
| Model 2 | Ref. | 1.02 (0.91-1.15) | 1.02 (0.90-1.17) | 1.21 (1.03-1.42) | 0.009 | Ref. | 0.79 (0.70-0.88) | 0.79 (0.69-0.90) | 0.75 (0.64-0.90) | <0.001 |
| Model 3 | Ref. | 0.98 (0.87-1.10) | 0.94 (0.82-1.10) | 1.01 (0.86-1.19) | 0.91 | Ref. | 0.80 (0.72-0.90) | 0.82 (0.72-0.93) | 0.78 (0.69-0.90) | 0.001 |
| **Male** | | | | | | | | | | |
| Model 1 | Ref. | 1.01 (0.90-1.12) | 1.03 (0.91-1.16) | 1.17 (1.01-1.15) | 0.020 | Ref. | 0.88 (0.79-0.97) | 0.84 (0.74-0.95) | 0.90 (0.78-1.04) | 0.014 |
| Model 2 | Ref. | 0.99 (0.89-1.10) | 0.99 (0.88-1.13) | 1.09 (0.94-1.26) | 0.21 | Ref. | 0.90 (0.81-0.99) | 0.86 (0.75-0.98) | 0.92 (0.79-1.06) | <0.001 |
| Model 3 | Ref. | 0.95 (0.85-1.06) | 0.92 (0.81-1.04) | 0.94 (0.81-1.09) | 0.40 | Ref. | 0.90 (0.81-1.00) | 0.87 (0.76-0.99) | 0.91 (0.79-1.05) | 0.042 |

**Model 1** was adjusted for age at baseline (continuous, years), race/ethnicity (White, non-White, and unknown), educational level (<9, 9 to<12, 12 to<16, ≥16 years, and unknown), Townsend deprivation index (continuous), and height (continuous, cm).

**Model 2** was further adjusted for smoking status (never, former, current: <10, 10 to<50, ≥50 pack-years, and unknown), drinking status (never, former, current: 0, <0.5, 0.5 to <1, ≥1 drinks/day, and unknown), sedentary time (continuous, h/day), and physical activity (continuous, MET-h/week).

**Model 3** was further adjusted for use of antihypertensive medication (yes, no), lipid-lowering medication (yes, no), diabetes medication (yes, no), and NSAIDs (yes, no).

Waist circumference and hip circumference were mutually adjusted for each other (in quartile).

**Table S4.** Associations of waist or hip circumference with all-cause dementia after excluding participants with sex-specific top or bottom 5% of waist or hip circumference.

|  | **Waist circumference** | | | | ***P*-trend** | **Hip circumference** | | | | ***P*-trend** |
| --- | --- | --- | --- | --- | --- | --- | --- | --- | --- | --- |
|  | **Q1** | **Q2** | **Q3** | **Q4** |  | **Q1** | **Q2** | **Q3** | **Q4** |  |
| **Female** | | | | | | | | | | |
| Model 1 | Ref. | 1.10 (0.97-1.24) | 1.08 (0.94-1.24) | 1.29 (1.11-1.51) | 0.001 | Ref. | 0.87 (0.77-0.97) | 0.84 (0.74-0.96) | 0.79 (0.68-0.91) | 0.001 |
| Model 2 | Ref. | 1.08 (0.95-1.23) | 1.05 (0.92-1.20) | 1.22 (1.05-1.43) | 0.012 | Ref. | 0.87 (0.77-0.97) | 0.85 (0.74-0.96) | 0.78 (0.67-0.91) | 0.001 |
| Model 3 | Ref. | 1.05 (0.92-1.19) | 0.97 (0.85-1.11) | 1.04 (0.89-1.22) | 0.78 | Ref. | 0.88 (0.78-0.99) | 0.87 (0.77-0.99) | 0.82 (0.70-0.96) | 0.008 |
| **Male** | | | | | | | | | | |
| Model 1 | Ref. | 1.05 (0.94-1.18) | 1.07 (0.94-1.21) | 1.12 (0.98-1.30) | 0.036 | Ref. | 0.89 (0.80-0.99) | 0.88 (0.77-0.99) | 0.87 (0.75-1.01) | <0.001 |
| Model 2 | Ref. | 1.04 (0.92-1.16) | 1.03 (0.91-1.18) | 1.06 (0.92-1.23) | 0.23 | Ref. | 0.90 (0.81-1.01) | 0.89 (0.79-1.01) | 0.89 (0.77-1.03) | 0.002 |
| Model 3 | Ref. | 1.00 (0.89-1.12) | 0.96 (0.85-1.10) | 0.94 (0.81-1.09) | 0.57 | Ref. | 0.91 (0.82-1.01) | 0.90 (0.79-1.02) | 0.90 (0.78-1.05) | 0.006 |

**Model 1** was adjusted for age at baseline (continuous, years), race/ethnicity (White, non-White, and unknown), educational level (<9, 9 to<12, 12 to<16, ≥16 years, and unknown), Townsend deprivation index (continuous), and height (continuous, cm).

**Model 2** was further adjusted for smoking status (never, former, current: <10, 10 to<50, ≥50 pack-years, and unknown), drinking status (never, former, current: 0, <0.5, 0.5 to <1, ≥1 drinks/day, and unknown), sedentary time (continuous, h/day), and physical activity (continuous, MET-h/week).

**Model 3** was further adjusted for use of antihypertensive medication (yes, no), lipid-lowering medication (yes, no), diabetes medication (yes, no), and NSAIDs (yes, no).

Waist circumference and hip circumference were mutually adjusted for each other (in quartile).

**Table S5.** Associations of predicted waist circumference or predicted hip circumference with all-cause dementia.

|  | **Waist circumference** | | | | ***P*-trend** | **Hip circumference** | | | | ***P*-trend** |
| --- | --- | --- | --- | --- | --- | --- | --- | --- | --- | --- |
|  | **Q1** | **Q2** | **Q3** | **Q4** |  | **Q1** | **Q2** | **Q3** | **Q4** |  |
| **Female** | | | | | | | | | | |
| Model 1 | Ref. | 0.95 (0.85-1.07) | 0.99 (0.88-1.11) | 1.20 (1.08-1.33) | <0.001 | Ref. | 0.82 (0.74-0.89) | 0.82 (0.74-0.90) | 0.81 (0.73-0.90) | <0.001 |
| Model 2 | Ref. | 0.96 (0.85-1.07) | 0.98 (0.88-1.10) | 1.16 (1.04-1.29) | <0.001 | Ref. | 0.82 (0.75-0.91) | 0.83 (0.75-0.92) | 0.81 (0.73-0.90) | <0.001 |
| Model 3 | Ref. | 0.95 (0.84-1.07) | 0.95 (0.85-1.06) | 1.05 (0.95-1.18) | 0.09 | Ref. | 0.85 (0.77-0.94) | 0.86 (0.78-0.96) | 0.83 (0.75-0.92) | <0.001 |
| **Male** | | | | | | | | | | |
| Model 1 | Ref. | 0.98 (0.88-1.10) | 1.02 (0.92-1.13) | 1.16 (1.05-1.28) | <0.001 | Ref. | 0.89 (0.81-0.97) | 0.85 (0.77-0.93) | 0.86 (0.78-0.96) | <0.001 |
| Model 2 | Ref. | 0.97 (0.87-1.09) | 1.00 (0.90-1.11) | 1.10 (1.00-1.22) | 0.007 | Ref. | 0.91 (0.84-0.99) | 0.88 (0.80-0.96) | 0.89 (0.80-0.98) | <0.001 |
| Model 3 | Ref. | 0.95 (0.85-1.06) | 0.95 (0.86-1.06) | 0.99 (0.89-1.09) | 0.70 | Ref. | 0.95 (0.87-1.04) | 0.92 (0.83-1.01) | 0.92 (0.83-1.02) | 0.005 |

**Model 1** was adjusted for age at baseline (continuous, years), race/ethnicity (White, non-White, and unknown), educational level (<9, 9 to<12, 12 to<16, ≥16 years, and unknown), Townsend deprivation index (continuous), and height (continuous, cm).

**Model 2** was further adjusted for smoking status (never, former, current: <10, 10 to<50, ≥50 pack-years, and unknown), drinking status (never, former, current: 0, <0.5, 0.5 to <1, ≥1 drinks/day, and unknown), sedentary time (continuous, h/day), and physical activity (continuous, MET-h/week).

**Model 3** was further adjusted for use of antihypertensive medication (yes, no), lipid-lowering medication (yes, no), diabetes medication (yes, no), and NSAIDs (yes, no).

Waist circumference and hip circumference were mutually adjusted for each other (in quartile).

**Table S6.** Associations of waist circumference with all-cause dementia across different age groups.

|  | **<50 y (n = 58,808)** | | | | ***P*-trend** | **≥50 y** **(n = 175,761)** | | | | ***P*-trend** |
| --- | --- | --- | --- | --- | --- | --- | --- | --- | --- | --- |
|  | **Q1** | **Q2** | **Q3** | **Q4** |  | **Q1** | **Q2** | **Q3** | **Q4** |  |
| **Female** | | | | | | | | | | |
| Model 1 | Ref. | 1.55 (0.63, 3.79) | 3.21 (1.26, 8.23) | 2.73 (0.87, 8.57) | 0.048 | Ref. | 1.03 (0.92, 1.17) | 1.03 (0.90, 1.18) | 1.29 (1.11, 1.51) | 0.001 |
| Model 2 | Ref. | 1.56 (0.64, 3.83) | 3.14 (1.21, 8.10) | 2.47 (0.78, 7.79) | 0.08 | Ref. | 1.03 (0.91, 1.16) | 1.00 (0.88, 1.15) | 1.21 (1.03, 1.41) | 0.014 |
| Model 3 | Ref. | 1.52 (0.62, 3.74) | 2.99 (1.15, 7.74) | 2.08 (0.64, 6.71) | 0.14 | Ref. | 0.98 (0.87, 1.10) | 0.92 (0.80, 1.05) | 1.00 (0.85, 1.18) | 0.97 |
| **Male** | | | | | | | | | | |
| Model 1 | Ref. | 1.11 (0.50, 2.49) | 3.22 (1.39, 7.47) | 2.43 (0.85, 6.94) | 0.026 | Ref. | 1.01 (0.90, 1.12) | 1.00 (0.88, 1.13) | 1.17 (1.01, 1.35) | 0.028 |
| Model 2 | Ref. | 1.06 (0.47, 2.39) | 2.78 (1.19, 6.48) | 1.97 (0.68, 5.68) | 0.07 | Ref. | 0.99 (0.88, 1.10) | 0.97 (0.85, 1.10) | 1.09 (0.94, 1.26) | 0.26 |
| Model 3 | Ref. | 1.04 (0.46, 2.33) | 2.62 (1.12, 6.16) | 1.67 (0.57, 4.89) | 0.15 | Ref. | 0.95 (0.85, 1.06) | 0.90 (0.79, 1.02) | 0.94 (0.81, 1.09) | 0.33 |

**Model 1** was adjusted for age at baseline (continuous, years), race/ethnicity (White, non-White, and unknown), educational level (<9, 9 to<12, 12 to<16, ≥16 years, and unknown), Townsend deprivation index (continuous), and height (continuous, cm).

**Model 2** was further adjusted for smoking status (never, former, current: <10, 10 to<50, ≥50 pack-years, and unknown), drinking status (never, former, current: 0, <0.5, 0.5 to <1, ≥1 drinks/day, and unknown), sedentary time (continuous, h/day), and physical activity (continuous, MET-h/week).

**Model 3** was further adjusted for use of antihypertensive medication (yes, no), lipid-lowering medication (yes, no), diabetes medication (yes, no), and NSAIDs (yes, no).

Waist circumference and hip circumference were mutually adjusted for each other (in quartile).

**Table S7.** Associations of hip circumference with all-cause dementia across different age groups.

|  | **<50 y (n = 51,364)** | | | | ***P*-trend** | **≥50 y (n = 154,928)** | | | | ***P*-trend** |
| --- | --- | --- | --- | --- | --- | --- | --- | --- | --- | --- |
|  | **Q1** | **Q2** | **Q3** | **Q4** |  | **Q1** | **Q2** | **Q3** | **Q4** |  |
| **Female** | | | | | | | | | | |
| Model 1 | Ref. | 1.04 (0.46, 2.38) | 0.76 (0.29, 1.99) | 0.68 (0.23, 2.01) | 0.29 | Ref. | 0.78 (0.70, 0.87) | 0.77 (0.68, 0.88) | 0.75 (0.65, 0.87) | <0.001 |
| Model 2 | Ref. | 1.06 (0.46, 2.43) | 0.79 (0.29, 2.08) | 0.71 (0.24, 2.11) | 0.33 | Ref. | 0.78 (0.70, 0.88) | 0.78 (0.68, 0.89) | 0.75 (0.64, 0.87) | <0.001 |
| Model 3 | Ref. | 1.05 (0.62, 3.74) | 0.78 (0.30, 2.08) | 0.70 (0.24, 2.09) | 0.34 | Ref. | 0.79 (0.71, 0.89) | 0.81 (0.71, 0.92) | 0.77 (0.66, 0.89) | 0.001 |
| **Male** | | | | | | | | | | |
| Model 1 | Ref. | 0.50 (0.23, 1.10) | 0.56 (0.23, 1.36) | 0.35 (0.12, 1.00) | 0.023 | Ref. | 0.88 (0.80, 0.98) | 0.83 (0.73, 0.95) | 0.90 (0.78, 1.05) | 0.014 |
| Model 2 | Ref. | 0.53 (0.24, 1.16) | 0.63 (0.26, 1.53) | 0.39 (0.14, 1.15) | 0.046 | Ref. | 0.90 (0.81, 0.99) | 0.85 (0.81, 0.97) | 0.92 (0.79, 1.06) | 0.029 |
| Model 3 | Ref. | 0.52 (0.23, 1.14) | 0.62 (0.25, 1.51) | 0.29 (0.13, 4.89) | 0.049 | Ref. | 0.90 (0.81, 1.00) | 0.86 (0.76, 0.98) | 0.90 (0.78, 1.05) | 0.09 |

**Model 1** was adjusted for age at baseline (continuous, years), race/ethnicity (White, non-White, and unknown), educational level (<9, 9 to<12, 12 to<16, ≥16 years, and unknown), Townsend deprivation index (continuous), and height (continuous, cm).

**Model 2** was further adjusted for smoking status (never, former, current: <10, 10 to<50, ≥50 pack-years, and unknown), drinking status (never, former, current: 0, <0.5, 0.5 to <1, ≥1 drinks/day, and unknown), sedentary time (continuous, h/day), and physical activity (continuous, MET-h/week).

**Model 3** was further adjusted for use of antihypertensive medication (yes, no), lipid-lowering medication (yes, no), diabetes medication (yes, no), and NSAIDs (yes, no).

Waist circumference and hip circumference were mutually adjusted for each other (in quartile).

**Table S8.** Associations of android or gynoid fat percent with brain structural measurements among females and males.

|  | **% Android fat** | | **% Gynoid fat** | |
| --- | --- | --- | --- | --- |
|  | female | male | female | male |
| **WMHV** |  |  |  |  |
| Model 1 | 4.24 (3.55, 4.92) | 6.23 (5.45, 7.02) | -2.91 (-3.43, -2.38) | -3.88 (-4.69, -3.07) |
| Model 2 | 3.89 (3.18, 4.59) | 5.88 (5.08, 6.69) | -2.69 (-3.22, -2.16) | -3.63 (-4.45, -2.82) |
| Model 3 | 2.80 (2.09, 3.51) | 4.60 (3.79, 5.42) | -1.96 (-2.50, -1.43) | -2.57 (-3.39, -1.76) |
| **TBV** |  |  |  |  |
| Model 1 | -0.52 (-1.18, 0.14) | -2.79 (-3.50, -2.07) | 0.93 (0.43, 1.44) | 1.32 (0.58, 2.05) |
| Model 2 | -0.61 (-1.28, 0.06) | -2.40 (-3.13, -1.68) | 1.06 (0.55, 1.57) | 1.19 (0.46, 1.93) |
| Model 3 | -0.32 (-1.01, 0.36) | -1.65 (-2.39, -0.91) | 0.89 (0.37, 1.41) | 0.56 (-0.18, 1.30) |
| **GMV** |  |  |  |  |
| Model 1 | -1.26 (-1.86, -0.66) | -3.77 (-4.43, -3.12) | 1.49 (1.03, 1.95) | 1.88 (1.20, 2.56) |
| Model 2 | -1.33 (-1.94, -0.71) | -3.51 (-4.18, -2.84) | 1.59 (1.13, 2.05) | 1.78 (1.10, 2.45) |
| Model 3 | -0.72 (-1.34, -0.09) | -2.53 (-3.21, -1.84) | 1.21 (0.74, 1.68) | 0.96 (0.28, 1.64) |
| **WMV** |  |  |  |  |
| Model 1 | 0.56 (-0.21, 1.33) | -0.54 (-1.39, 0.31) | -0.08 (-0.67, 0.51) | 0.14 (-0.73, 1.01) |
| Model 2 | 0.48 (-0.31, 1.27) | -0.17 (-1.04, 0.70) | 0.03 (-0.57, 0.63) | 0.04 (-0.83, 0.91) |
| Model 3 | 0.27 (-0.54, 1.07) | 0.02 (-0.86, 0.91) | 0.17 (-0.44, 0.77) | -0.14 (-1.02, 0.75) |
| **HV** |  |  |  |  |
| Model 1 | -0.97 (-1.68, -0.26) | -1.09 (-1.97, -0.22) | 0.57 (0.02, 1.11) | 0.37 (-0.52, 1.27) |
| Model 2 | -0.63 (-1.36, 0.09) | -0.79 (-1.68, 0.11) | 0.38 (-0.16, 0.93) | 0.24 (-0.66, 1.13) |
| Model 3 | -0.21 (-0.95, 0.53) | -0.48 (-1.40, 0.43) | 0.10 (-0.45, 0.66) | -0.03 (-0.94, 0.88) |
| **HGMV** |  |  |  |  |
| Model 1 | -1.86 (-2.56, -1.16) | -1.90 (-2.71, -1.09) | 1.10 (0.56, 1.63) | 0.56 (-0.27, 1.40) |
| Model 2 | -1.66 (-2.38, -0.94) | -1.72 (-2.55, -0.89) | 0.96 (0.42, 1.51) | 0.46 (-0.38, 1.29) |
| Model 3 | -1.36 (-2.09, -0.63) | -1.46 (-2.31, -0.61) | 0.76 (0.21, 1.31) | 0.20 (-0.65, 1.05) |

**Model 1** was adjusted for age at DXA scan (continuous, years), race/ethnicity (White, non-White, and unknown), educational level (<9, 9 to<12, 12 to<16, ≥16 years, and unknown), Townsend deprivation index (continuous), and height (continuous, cm).

**Model 2** was further adjusted for smoking status (never, former, current: <10, 10 to<50, ≥50 pack-years, and unknown), drinking status (never, former, current: 0, <0.5, 0.5 to <1, ≥1 drinks/day, and unknown), and physical activity (continuous, MET-h/week).

**Model 3** was further adjusted for use of antihypertensive medication (yes, no), lipid-lowering medication (yes, no), diabetes medication (yes, no), and NSAIDs (yes, no).

GMV, grey matter volume; HGMV, hippocampal grey matter volume; HV, hippocampus volume; TBV, total brain volume; WMHV, white matter hyperintensity volume; WMV, white matter volume


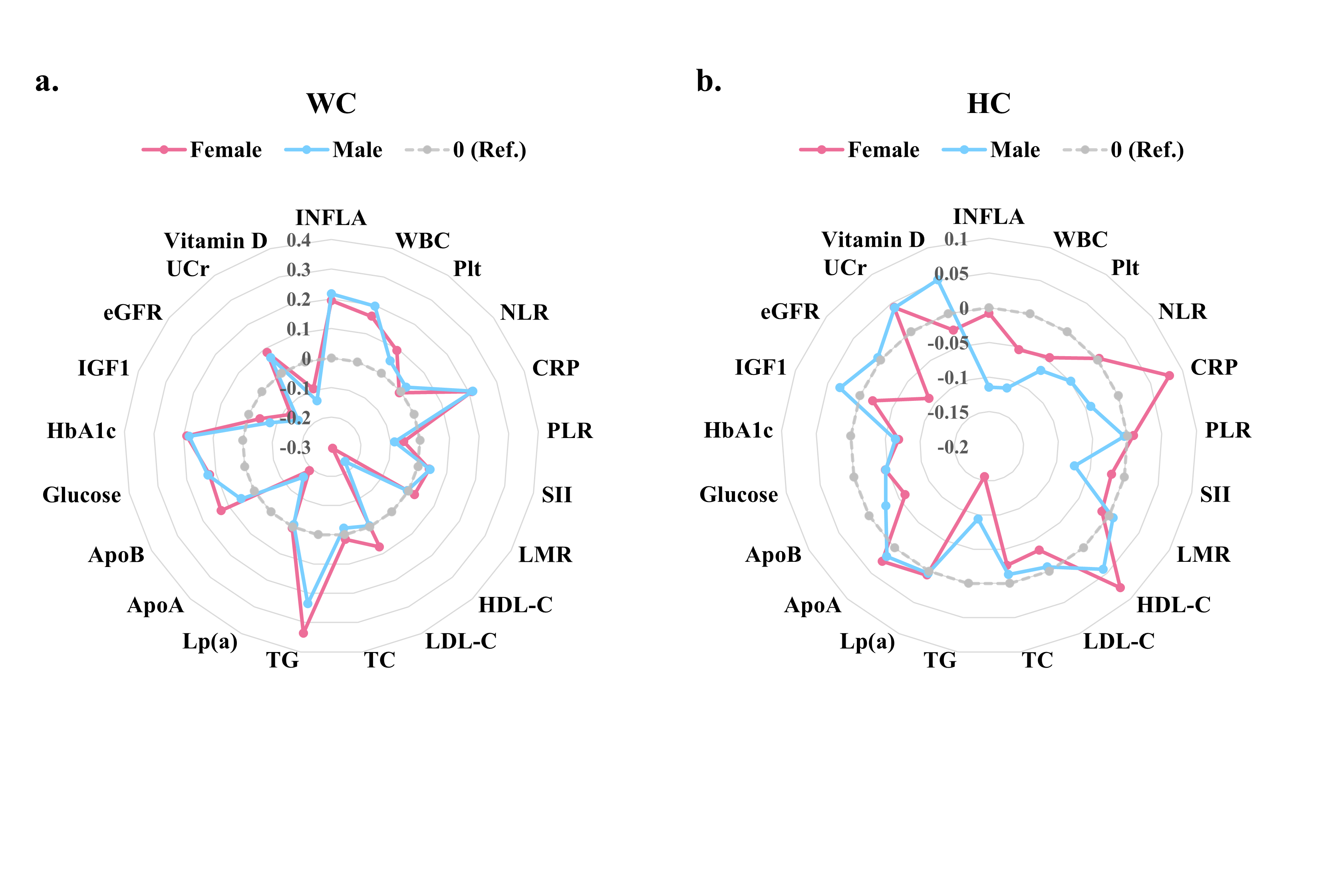


**Figure S1.** Correlations between waist or hip circumference and speculated potential mediators among females and males.

Results are partial Pearson correlation coefficients adjusted for age and race/ethnicity. WC and HC were mutually adjusted for each other as continuous variables.

ApoA, apolipoprotein A; ApoB, apolipoprotein B; CRP, high-sensitivity C-reactive protein; eGFR, estimated glomerular filtration rate; HbA1c, glycosylated hemoglobin; HC, hip circumference; HDL-C, high-density lipoprotein cholesterol; IGF 1, insulin-like growth factor 1; LDL-C, low-density lipoprotein cholesterol; LMR, lymphocyte to monocyte ratio; Lp(a), lipoprotein(a); NLR, neutrophil to lymphocyte ratio; PLR, platelet to lymphocyte ratio; Plt, platelet count; SII, systemic immune-inflammation index; TC, total cholesterol; TG, triglycerides; UCR, urinary creatinine; WBC, white blood cell count; WC, waist circumference
